# CARDIAC TROPONIN T MEDIATED AUTOIMMUNE RESPONSE AND ITS ROLE IN SKELETAL MUSCLE AGING

**DOI:** 10.1093/geroni/igz038.3231

**Published:** 2019-11-08

**Authors:** Tan Zhang, Xin Feng, Bo Feng, Juan Dong, Karen Haas, Barbara M Nicklas, Osvaldo Delbono, Stephen Kritchevsky

**Affiliations:** 1 Wake Forest School of Medicine, Winston-Salem, North Carolina, United States; 2 Wake Forest School of Medicine, Winston-Salem, United States; 3 Wake Forest University, Winston-Salem, North Carolina, United States

## Abstract

Cardiac troponin T (cTnT), a key component of contractile machinery essential for muscle contraction, is also expressed in skeletal muscle under certain conditions (e.g. neuromuscular diseases and aging). We have reported that skeletal muscle cTnT regulates neuromuscular junction denervation preferentially in fast skeletal muscle of old mice. Here, we further report that cTnT is also enriched within some myofibers, and/or along microvascular walls in old mice fast skeletal muscle. Strikingly, immunoglobulin G (IgG), together with markers of complement system activation, cell death (necroptosis or apoptosis), and macrophage infiltration, were all found to be co-localized with cTnT and IgG in those areas. In addition, elevated cTnT and IgG are associated with lower dystrophin expression on muscle fiber membrane, lower muscle capillary density, and reduced muscle performance (wire hanging test). Using purified recombinant TnT proteins, we confirmed that only cTnT, but not slow or fast skeletal muscle TnT1 or TnT3, was detected by immunoblot using sera from old (but not young) mice with pre-determined elevated cTnT and IgG in their skeletal muscle, indicating the existence of anti-cTnT autoantibodies in sera (previously found in human blood) and skeletal muscle of old mice. Immunoblotting further revealed that the age related changes in skeletaI muscle cTnT and IgG are more prominent in fast skeletal muscle than in slow. Importantly, elevated cTnT and IgG were also detected in skeletal muscles from 4 older adults (65-70 yrs, IMFIT). Our finding suggests a novel autoimmune mechanism mediated by cTnT that underlies age related skeletal muscle abnormalities and dysfunction.

